# Oral Functions Are Associated with Muscle Strength and Physical Performance in Old-Old Japanese

**DOI:** 10.3390/ijerph182413199

**Published:** 2021-12-15

**Authors:** Yuki Murotani, Kodai Hatta, Toshihito Takahashi, Yasuyuki Gondo, Kei Kamide, Mai Kabayama, Yukie Masui, Tatsuro Ishizaki, Ken-ichi Matsuda, Yusuke Mihara, Motoyoshi Fukutake, Yuichi Nishimura, Suzuna Akema, Hiromasa Hagino, Kotaro Higashi, Hitomi Togawa, Yoshinobu Maeda, Soshiro Ogata, Paula Moynihan, Kazunori Ikebe

**Affiliations:** 1 Department of Prosthodontics, Gerodontology and Oral Rehabilitation, Osaka University Graduate School of Dentistry, Suita 565-0871, Japan; y-murotani@dent.osaka-u.ac.jp (Y.M.); toshi-t@dent.osaka-u.ac.jp (T.T.); digiflex@dent.osaka-u.ac.jp (K.-i.M.); y-mihara@dent.osaka-u.ac.jp (Y.M.); m-fukutake@dent.osaka-u.ac.jp (M.F.); y-nishimura@dent.osaka-u.ac.jp (Y.N.); akema@dent.osaka-u.ac.jp (S.A.); h-hagino@dent.osaka-u.ac.jp (H.H.); higashi@dent.osaka-u.ac.jp (K.H.); ymaeda@dent.osaka-u.ac.jp (Y.M.); ikebe@dent.osaka-u.ac.jp (K.I.); 2 Department of Clinical Thanatology and Geriatric Behavioral Science, Osaka University Graduate School of Human Sciences, Suita 565-0871, Japan; ygondo@hus.osaka-u.ac.jp; 3 Division of Health Sciences, Osaka University Graduate School of Medicine, Suita 565-0871, Japan; kamide@sahs.med.osaka-u.ac.jp; 4 Integrated General Nursing Science, Graduate School of Medicine, Osaka University, Suita 565-0871, Japan; kabayama@sahs.med.osaka-u.ac.jp; 5 Tokyo Metropolitan Geriatric Hospital and Institute of Gerontology, Tokyo 173-0015, Japan; yukie@krf.biglobe.ne.jp (Y.M.); tatsuro@tmig.or.jp (T.I.); 6 Division of Comprehensive Prosthodontics, Niigata University Graduate School of Medical and Dental Sciences, Niigata 951-8514, Japan; h-togawa@dent.niigata-u.ac.jp; 7 National Cerebral and Cardiovascular Center, Department of Preventive Medicine and Epidemiology, Suita 564-8565, Japan; s_ogata@ncvc.go.jp; 8 Faculty of Health and Medical Sciences, Adelaide Dental School, The University of Adelaide, Adelaide, SA 5005, Australia; paula.moynihan@adelaide.edu.au

**Keywords:** oral health, hand strength, physical functional performance

## Abstract

Grip strength and walking speed are considered to be important indicators of physical frailty. However, no study has contemporaneously examined any association of multiple oral functions with grip strength and walking speed. The purpose of this study was to examine which oral functions are associated with muscle strength (grip strength), physical performance (walking speed) or both. The study participants were 511 community-dwelling people (254 men and 257 women) aged 77–81 years old. Six oral functions—oral wetness, occlusal force, tongue-lip motor function, tongue pressure, masticatory performance and swallowing function—were measured. Grip strength and walking speed were also measured. A partial correlation analysis, adjusted for gender, showed that occlusal force, tongue-lip motor function, masticatory performance and swallowing function were significantly associated with both grip strength and walking speed. In addition, tongue pressure was significantly associated with grip strength. A general linear model showed that tongue pressure and occlusal force were significantly associated with grip strength. Swallowing function and tongue-lip motor function were significantly associated with walking speed. It is suggested that there are different oral function measures for muscle strength and physical performance, and these oral function measures could be a useful proxy for physical frailty.

## 1. Introduction

Grip strength and walking speed are considered to be important indicators of physical decline for older people to live independently [1,2,3]. Grip strength is used as an index of muscle strength and walking speed is used as an index of physical performance in the diagnosis of physical frailty [4,5]. Physical frailty is considered to be one of the main causes of dependency and has been recognized as an important priority in Japan, where there is an overall pooled prevalence among community-dwelling older people of 7.4% [6]. Therefore, the early detection of the signs of physical frailty is of paramount importance.

Previous studies have reported on the relationships between hand grip strength and individual measures of oral health measures including occlusal force [7,8], tongue pressure [8,9], masticatory performance [8,10] and swallowing function [8]. Previous studies have also explored the relationship between walking speed and occlusal force [11] and masticatory performance [12]. From these reports, it is considered that oral function measures could be a good proxy for a risk of physical frailty and could be used to spot those at risk. However, no study has examined contemporaneously any associations of multiple oral functions with grip strength and walking speed in older adults.

The purpose of this study was to examine which oral functions are associated with muscle strength (grip strength), physical performance (walking speed) or both and the relative size and strength of any associations in old-old Japanese. The overall aim was to identify the best oral health proxy measures of physical frailty that can be applied in dental practice.

## 2. Materials and Methods

The study protocol was approved by the Institutional Review Board of the Osaka University Graduate School of Dentistry (approval number H27-E4). This article was prepared in accordance with the STROBE statement [13].

### 2.1. Participants

The study population was drawn from the SONIC (the Septuagenarians, Octogenarians, Nonagenarians Investigation with Centenarians) study. The participants were community-dwelling, independently living people. They were volunteers from two regions of eastern and western Japan (the Tokyo Metropolitan and the Hyogo Prefecture, respectively). An urban area and a rural area were included in both regions for a total of four geographic areas: Itami City, Hyogo (Western urban); Asago City, Hyogo (Western rural); Itabashi Ward, Tokyo (Eastern urban); and Nishitama Country, Tokyo (Eastern rural). Community-dwelling older residents in each area were identified from the local residential register and contacted by mail. We sent invitation letters to 1229 residents to participate in this study. Ultimately, 550 volunteers participated in the survey from July 2019 to December 2019. The inclusion criteria were: (a) all participants could complete the oral functions tests; and (b) all participants could complete the physical tests. Those with missing values were excluded. 

All participants in this study gave written informed consent to participate.

### 2.2. Measurements

#### 2.2.1. Oral Functions

All participants were examined for six oral functions by registered dentists who were trained in the examination methods used in this study. Participants with removable partial or complete dentures kept their dentures in place during the measurements of oral functions.

Oral wetness was measured with an oral moisture checker (Mucus; Life, Saitama, Japan). Mucosal wetness was measured in the central area of the tongue dorsum. Oral wetness measurements were performed three times and the median was used in the analysis [14,15].

The bilateral maximal occlusal force was measured using pressure-sensitive sheets (Dental Prescale 50H R type; GC, Tokyo, Japan). The participants performed maximal clenching in the intercuspal position with the sheet placed between their upper and lower dental arches. The maximal occlusal force (Newton (N)) was calculated after scanning the sheet with an image scanner (Occluzer FDP709; GC, Tokyo, Japan) [16].

Tongue-lip motor function was recorded using an automatic counter (Kenkokun Handy; Takei Scientific Instruments, Niigata, Japan). The participants pronounced each word, /pa/, /ta/ and /ka/, clearly and repeatedly as fast as possible for five seconds. The number of times the participant could pronounce these in one second was counted as the outcome variable for use in the analysis [17,18,19].

Tongue pressure was measured using a tongue pressure measurement device (JMS tongue pressure measuring instrument TPM-01; JMS, Hiroshima, Japan). The participants were asked to sit in a relaxed position. They were then instructed to hold the plastic probe in their central incisors with their lips closed whilst compressing the balloon between their tongue and their hard palate for five seconds with a maximal voluntary effort. Tongue pressure measurements were performed three times and the average value was used in the analysis [20].

Masticatory performance was measured using a piece of gummy jelly (Test gummy jelly; UHA Mikakuto, Osaka, Japan). The participants were instructed to freely masticate the gummy jelly 30 times without swallowing. Comminuted gummy jellies were compared with images of visual materials that were scored on a scale of 0–9 [21].

A Repetitive Saliva Swallowing Test (RSST) was used to assess swallowing ability. The participants were asked to sit in a relaxed position and perform repetitive voluntary swallowing as quickly as possible for 30 s. The number of swallowings was determined by palpation of the laryngeal movement with the examiner’s second and third fingers [18,22].

#### 2.2.2. Grip Strength and Walking Speed

Grip strength was evaluated as an index of muscle strength. Isometric grip strength was measured using a Smedley hand grip dynamometer (Model YD-100; Yagami Ltd., Tokyo, Japan) as an indicator of general muscle strength. The strength of the dominant hand was measured twice and the average was calculated (kg).

Walking speed was evaluated as an index of physical performance. The number of seconds taken to walk eight feet at a normal speed was measured twice and the two measurements were averaged to give the usual walking speed (meters/second (M/s)).

#### 2.2.3. Other Variables

The number of remaining natural teeth of each participant was recorded by a dentist using a dental mirror and an explorer. The researchers also obtained actual measurements of the heights of participants using a stadiometer with shoes off as a single measure.

### 2.3. Statistical Analysis

A Mann–Whitney U test was performed to investigate any between-gender differences in the outcome measures. A partial correlation analysis, adjusted for gender, was performed to investigate any association of oral functions with grip strength and walking speed. A general linear model was performed to investigate any association after adjusting for gender, the number of teeth and height. Each independent variable was standardized. The statistical analyses were conducted using SPSS statistics 25 (IBM Japan, Tokyo, Japan). The statistical significance level was set at 5%.

## 3. Results

Of the 550 participants, 546 participants underwent oral function tests, 548 participants underwent physical tests and 531 participants had their height measured. We excluded 39 participants with missing data (occlusal force: 8, masticatory performance: 2, tongue pressure: 4, grip strength: 1, walking speed: 4). Finally, 511 participants (men: 254, women: 257, aged 77–81) were included in the analysis.

Table 1 shows the median (IQR) values for the measures of oral function and physical frailty. The medians (and IQR) of each outcome variable are shown by gender. With the exception of tongue pressure and number of teeth, all outcome variables were significantly different between the men and women.

Table 2 shows the results of the partial correlation analysis. Gender was used as the control variable. As shown in Table 2, occlusal force, tongue-lip motor function (/ta/) and (/ka/), tongue pressure, masticatory performance and swallowing function were significantly associated with grip strength. Occlusal force, tongue-lip motor function (/pa/) and (/ta/), masticatory performance and swallowing function were significantly associated with walking speed.

Table 3 shows the results of the general linear model for grip strength. Occlusal force, tongue pressure and gender were significantly associated with grip strength after adjusting for other variables. The data in Table 3 also show that the strongest association was noted between tongue pressure and grip strength (β = 0.13).

Table 4 shows the results of the general linear model for walking speed. Tongue-lip motor function /pa/, swallowing function and gender were significantly associated with walking speed after adjusting for other variables. The data in Table 4 also show that the strongest association was noted between swallowing function and walking speed (β = 0.15).

## 4. Discussion

This study investigated the associations of oral functions with muscle strength and physical performance in community-dwelling older Japanese. The result showed that occlusal force and tongue pressure were significantly associated with grip strength. From the standardized regression coefficients, it was clear that tongue pressure was associated with grip strength most strongly. Tongue-lip motor function and swallowing function were significantly associated with walking speed. From the standardized regression coefficients, it was clear that swallowing was associated with walking speed most strongly.

These results showed that different oral function measures were associated with muscle strength from those associated with physical function. 

Iinuma et al. reported that a lower occlusal force increased the risk of grip strength decline only in men [7]. Mihara et al. reported that grip strength was significantly correlated with the maximum occlusal force (β = 0.20) and tongue pressure (β = 0.34) [10]. Similar to these previous studies, our study also found that grip strength was associated with occlusal force (β = 0.12) and tongue pressure (β = 0.13). This study confirms these previous studies.

Okada et al. reported that a slower walking speed was associated with a lower occlusal force [11] and Kamdem et al. reported that self-reported impaired masticatory ability was linked to a low gait speed [12]. In the present study, Table 2 shows that walking speed was significantly correlated with occlusal force. However, when adjusting for other oral functions, as shown in Table 4, occlusal force was not significantly associated with walking speed but tongue-lip motor function and swallowing function were significantly associated with walking speed. This suggests that complex functions such as tongue-lip motor function and swallowing function are more associated with walking speed than occlusal force.

To our knowledge, this is the first study that has enabled a comparison of the relative strength of the association of a number of oral health markers with the markers of physical frailty. This was achieved through the contemporaneous measurement of several distinct measures of oral function and muscle strength and physical performance on the same day and by entering values into one model as independent variables to evaluate the associations of oral function with both grip strength and walking speed.

The findings are biologically plausible because occlusal force, tongue pressure and grip strength are all exerted by muscle strength. On the other hand, swallowing, tongue-lip motor function and walking speed are all related to muscle function and may be interrelated [23]. The findings suggest that oral muscle strength is related to muscle strength (grip strength) and oral motor function is related to physical performance (walking speed) and that different oral function measures may be a useful proxy measure for either. Several limitations of this study should be acknowledged. First, the study population was narrow and included only non-clinical, non-institutionalized and community-dwelling Japanese people aged 77–81 years old. Although the sample was drawn from a complete enumeration of the resident records, most of the participants were physically healthy. In addition, oral wetness, occlusal force, tongue-lip motor function /pa/ and /ta/, masticatory performance, swallowing function and the number of remaining teeth were higher than the diagnostic criteria for oral hypofunction in Japanese people over 65 years old. The only measurement values that were lower were tongue-lip motor function /ka/and tongue pressure [22,24]. Therefore, there is a possibility that people with a severe physical decline and people who were uninterested in their health were likely excluded from this study. Consequently, the results cannot be generalized to younger or less healthy people. 

Second, the study was cross-sectional and thus it was not possible to determine whether a decline in oral health preceded a decline in physical health or vice versa or whether both occurred concurrently and, therefore, causality could not be determined through the association. Previous research has suggested that a decline in oral function impacts on diet including the intake of protein [25], which in turn could contribute to the development of frailty. However, any role of nutrition in mediating an association between oral health and physical frailty remains to be confirmed. Therefore, we considered that a decline in oral health affected physical health. However, it is necessary to follow older people longitudinally and identify any direction of effect. 

Third, due to the multiple study purposes (description of the ageing process and the identification of factors influencing healthy longevity) and the multidisciplinary nature of the variables collected in the SONIC study, it was not possible to calculate an appropriate sample size for the present study.

## 5. Conclusions

In community-dwelling older Japanese, occlusal force and tongue pressure were good indicators of muscle strength (grip strength). Tongue-lip motor function and swallowing function were good indicators of physical performance (walking speed). These oral function measures could be useful proxy measures for a physical decline in older people and may be useful in screening for physical frailty at a dental practice that warrants further exploration in longitudinal studies.

## Figures and Tables

**Table 1:** Median (IQR) values for the measures of oral function and physical frailty.

Survey Items	Median (IQR)				*p*-Value
	Men (*n* = 254)		Women (*n* = 257)		
Oral wetness	28.4	(26.4–30.1)	27.5	(25.4–29.4)	<0.01
Occlusal force (N)	342.0	(148.1–550.1)	272.4	(134.8–455.5)	0.02
Tongue-lip motor function /pa/ (times per second)	6.0	(5.2–6.5)	6.2	(5.8–6.6)	<0.01
Tongue-lip motor function /ta/	6.0	(5.2–6.4)	6.0	(5.4–6.6)	0.04
Tongue-lip motor function /ka/	5.4	(4.6–6.0)	5.6	(5.2–6.2)	<0.01
Tongue pressure (kPa)	27.1	(22.4–33.2)	27.2	(21.9–32.0)	0.63
Masticatory performance	6.0	(3.0–7.0)	5.0	(2.0–7.0)	0.01
Swallowing function	5.0	(4.0–6.0)	4.0	(2.0–5.0)	<0.01
Number of remaining teeth	23.0	(15.0–26.3)	22.0	(15.0–26.0)	0.32
Grip strength (kg)	30.0	(26.5–35.0)	20.0	(17.0–22.8)	<0.01
Walking speed (m/s)	0.97	(0.88–1.08)	1.03	(0.88–1.14)	0.01
Height (cm)	163.8	(159.4–167.2)	149.8	(146.5–153.1)	<0.01

IQR: interquartile range. *p*-values were determined using the Mann–Whitney U test.

**Table 2:** The results of the partial correlation analysis between the oral function and physical frailty measures.

Variables Control Variable: Gender	Grip Strength		Walking Speed	
	*r*	*p*-Value	*r*	*p*-Value
Oral wetness	0.02	0.70	−0.03	0.53
Occlusal force (N)	0.23	<0.01	0.10	0.02
Tongue-lip motor function /pa/ (times per second)	0.08	0.07	0.15	<0.01
Tongue-lip motor function /ta/	0.14	<0.01	0.10	0.02
Tongue-lip motor function /ka/	0.10	0.02	0.07	0.10
Tongue pressure (kPa)	0.26	<0.01	0.08	0.06
Masticatory performance	0.20	<0.01	0.10	0.03
Swallowing function	0.11	0.02	0.17	<0.01

N: Newton. *r*: partial correlation coefficient.

**Table 3:** The results of the general linear model for grip strength.

Independent Variables	β (95% CI)		*p*-Value
Gender (reference: men)	−0.90	(−1.08–−0.73)	<0.01
Height	0.31	(0.22–0.40)	<0.01
Oral wetness	0.01	(−0.04–0.07)	0.69
Occlusal force	0.12	(0.05–0.18)	<0.01
Tongue-lip motor function /ta/	0.05	(−0.004–0.11)	0.07
Tongue pressure	0.13	(0.07–0.18)	<0.01
Swallowing function	0.04	(−0.02–0.10)	0.16
Number of remaining teeth	0.01	(−0.06–0.07)	0.86

Dependent variable: grip strength (kg). β: standardized regression coefficient. CI: confidence interval.

**Table 4:** The results of the general linear model for walking speed.

Independent Variables	β (95% CI)		*p*-Value
Gender (reference: men)	0.56	(0.29–0.84)	<0.01
Height	0.21	(0.08–0.35)	<0.01
Oral wetness	−0.02	(−0.10–0.07)	0.67
Occlusal force	0.07	(−0.03–0.18)	0.17
Tongue-lip motor function /pa/	0.12	(0.03–0.21)	0.01
Tongue pressure	0.03	(−0.05–0.12)	0.46
Swallowing function	0.15	(0.06–0.24)	<0.01
Number of remaining teeth	−0.02	(−0.13–0.08)	0.68

Dependent variable: walking speed (m/s). β: standardized regression coefficient. CI: confidence interval.

## Data Availability

The data presented in this study are available on request from the corresponding author. The data are not publicly available due to privacy.
